# Supplementary material for: Illuminating biological pathways for drug targeting in head and neck squamous cell carcinoma
Source: PLoS One. 2019 Oct 9;14(10):e0223639. doi: 10.1371/journal.pone.0223639 (PMC6785123; doi:10.1371/journal.pone.0223639)
Supplement: Attachment — Submitted filename: Choonoo_etal_ResponseToReviewers_091619.pdf [file pone.0223639.s009.pdf]

PONE-D-19-19753
Illuminating Biological Pathways for Drug Targeting in Head and Neck Squamous Cell Carcinoma
Response to Reviewers

ACADEMIC EDITOR: The authors pull data from available public head and neck squamous cell carcinoma sequencing and cancer drug databases to characterize "light" and "dark" pathways, highlighting opportunities for drug development in "dark" pathways. Overall, there are interesting concepts in the manuscript, but it needs significant revision. I agree with Reviewer 2 that the analysis without copy number variation significantly limits the impact of the work. A significant amount of mutations in HNSCC are CNVs; indeed EGFR, one of the most established aberrant genes in HNSCC and the target of cetuximab (as the authors note), is not pulled out by this analysis as its alterations generally are of CNV and expression changes. Also PIK3CA, as the researchers highlight, has significant CNV aberrations. Similar research by other groups often includes CNV. I would recommend the authors include CNV/expression analysis, or describe their rationale otherwise. Additionally, as Reviewer 2 notes, there has been a fair amount of previous research in this topic. The authors do have potentially novel angles to explore, but should do so in the context of discussions on previous approaches and how the approaches of the authors are unique and add novelty.

The authors should address address major comments 2-4 by reviewer 2. Addressing major comment 1 by reviewer 2 would significantly strengthen the author's research if possible.

Thank you for your comments. For major comment 1 by reviewer 2, we have provided more discussion with regard to the novelty and advantages of our approach. Given that this work as presented here is solely computational and focused on the pathway framework, functional validation is currently out of scope for this manuscript. Our group is actively working on integrating these pathway-based genomic data analyses with functional analyses, including patient-derived tumor cell responses to a custom designed, high throughout drug screening panel in process. These aspects will be addressed in future manuscripts in context of the current work.

We have addressed points 2-4 by reviewer 2. In particular, we agree with Reviewer 2 with regard to the inclusion of copy number. We have

incorporated copy number alterations into our pathway analysis and believe that it has significantly strengthened our manuscript. Unfortunately, inclusion of expression data for this project is currently out of scope. Our group (McWeeney) has developed outlier approaches for individual patient expression (Bottomly et al 2013) and recognizes the inclusion and integration of individual level individual patient expression estimates is nontrivial. However our approaches and the dataset we share in the current work provide a basis for inclusion of expression data into these workflows in the future by us and others and a rich area for methodology development.

Finally, we have made revisions throughout the paper to strengthen discussion of previous approaches and compare and contrast these with our own work.

Reviewer #1: Here, the authors present a timely analysis correlating sequencing data of head and neck cancer subtypes mutational loads with its associated "drugome". The authors submit robust analyses, which well articulate their workflow, results, and significance of their work. I anticipate these data are of high value to otolayrngologists, and translational researchers developing drugs for head and neck cancers. Such work warrants a commentary piece in this reviewer's opinion.

Thank you for this recommendation.

Reviewer #2: The authors use a systems biology approach with publicly available data to discern "novel" drug targets in both HPV negative and HPV positive HNSCC based on genetics and drug binding data. Overall, this manuscript has compiled a fairly superficial analysis of public data, which, at first appearance, has already been completed in a similar fashion by other groups, and which is limited to just somatic mutations. In this case, the team applies a unique statistical approach to highlight potential pathways as light or dark, and chose to highlight the PI3K pathway as evidence that the approach was successful (which is already being evaluated in HNSCC clinical trials).

Major comments:

1) Demonstrate that this approach has novelty and can make an impact. The authors need to perform some functional validation, or use data from publicly available drug testing databases, in HNSCC models, to show that this

approach adds to existing approaches already used in the literature.

We appreciate the opportunity to address this important comment. We have added to our discussion to specifically address the novelty of our approach. Given the limited treatment options currently available for HNSCC, our work presented here is primarily focused on increasing the potentially targetable space for head and neck cancer, a cancer with few options. The scope of this paper is to present our computational contribution to expanding the potentially targetable space, and has implications for other cancers impacted by similar pathways. Indeed, our work here is intended to be incorporated into larger pipelines (such as drug screening panels) to identify targetable disease-specific vulnerabilities. As noted above, our group is active in designing and applying such a screening panel for patient-derived head and neck cancer cells and PDX models.

We have presented our computational approach for pathway analysis as we believe it is of interest to the wider precision medicine community. We point out that it is in line with the PLOS Call for Papers, under area #2: “Target and drug discovery”, which indicated:

> “We welcome manuscript reporting research in all areas of target and drug discovery, including: computational chemoinformatic and/or machine learning approaches to finding ‘druggable’ targets as well as identifying novel compounds”

We felt this would be an excellent opportunity to showcase our computational approach as well as make our methods open and reproducible for the community. Given that functional validation of the results presented here can take many approaches, we feel strongly that submission of our computational approach via this call for papers will provide a basis for active dissemination and discussion, and follow-up validation on multiple fronts, besides our own.

2) Enhance the discussion to compare and contrast this approach to similar previous approaches, reference existing literature on the topic.

We have added substantial text to the introduction and discussion to address previous related work in HNSCC in a deeper manner with accompanying references. We have provided further discussion comparing and contrasting our pathway approach with other methods. One key example, is that, while

other approaches have investigated pathway level aberrations, our approach unites patient-level pathway alterations with drug-target information in a much more comprehensive manner. Typically, approaches focus on drug-target interactions only for a subset of target interactions – “primary” targets or classes/families of targets. Here, we use all known drug-target interactions with supportive evidence in the Cancer Targetome in an effort to substantially expand the targetable pathway space in HNSCC. Additionally, our work offers both potentially targetable space (light pathways) as well as currently un-targeted space (dark pathways), which we believe provides a framework to the HNSCC research community for consideration of opportunities for repurposing of available drugs and/or development of novel therapeutics.

Additionally, our methodology leverages all open source resources, such as the Reactome pathway database for biological pathway annotation and our own Cancer Targetome resource for drug-target interactions. We have made our methods and pipeline both accessible and reproducible for use in other applications by the community, see our GitHub page: https://github.com/biodev/HNSCC_Notebook

3) Why did the authors chose to ignore copy number and/or expression data when designing this approach? Other approaches have successfully implemented additional data for lead target optimization.

Thank you for bringing up this point. At the reviewer and editor’s suggestion, we have incorporated additional pathway analysis for copy number for the HNSCC cohort. Given that recurrent copy number alterations have previously been found to be an important aspect of HNSCC dysregulated signaling, we agree that this should be included in our analysis. Please see the following section – “HNSCC Copy Number Alterations” for discussion of specific copy number alterations in this cohort. Additionally, the section “HNSCC Light and Dark Pathways” now includes analysis of pathways enriched for copy number alterations and/or drug-targeted.

4) The writing needs to be improved. The authors only have 20 references and simply gloss over important references and details related to this work, for example, in this section "Targeting the PI3K pathway with PI3K inhibitors in enriched tumors was effective in vitro and in vivo". No references.

We have extensively revised the manuscript to address clarity and context of the literature, incorporating relevant citations throughout the body of the manuscript. (The sentence pointed out above the by the reviewer was immediately followed by another sentence citing the appropriate literature reference but we agree it was not clear. We have now included the citation in both sentences for clarity.)

Minor comments:

1) Figure quality could be improved substantially, especially the mutation rate figure.

While it is a little unclear from this feedback if we have captured all aspects of Figure 2 that need to be improved, we have modified the subtitles and axes labels (larger, size, bolder) in order to address this issue. Additionally, Figure 2 has now been expanded to include 2A for mutation data and 2B for copy number data. We have also expanded Figure 3 to reflect both mutation and copy number enriched pathways and added a new figure (Figure 4) to highlight light and dark pathway subsets of interest.
